# Post-mortem urine dipstick analysis for hyperglycemia and ketoacidosis: observer agreement and diagnostic value

**DOI:** 10.1007/s00414-025-03477-3

**Published:** 2025-04-03

**Authors:** Joanna M. Glengarry, Ben Thompson, Maria Pricone, Melanie S. Archer, Hans H. de Boer

**Affiliations:** 1 Victorian Institute of Forensic Medicine, 65 Kavanagh St, Southbank, VIC 3006 Australia; 2 Department of Forensic Medicine, Monash University, Southbank, VIC 3006 Australia

**Keywords:** Acetone, Autopsy, Biochemistry, Dipstick, Forensic medicine, Forensic pathology, Glucose, Hyperglycemia, Ketones, BHB, Point of care testing, Post-mortem, Urinalysis, Urine

## Abstract

The post-mortem diagnosis of hyperglycemia and/or ketoacidosis is challenging and usually requires costly ancillary testing of vitreous humor or serum samples. A screening tool that would help to determine whether ancillary testing is needed is therefore desirable. The aim of this study was to add to the literature testing the validity and diagnostic utility of post-mortem dipstick urinalysis. More specifically, we determined inter-observer agreement of visual dipstick assessment, the correlation between glucose and ketone urine dipstick scores and formal laboratory testing results, and the diagnostic value of specific dipstick scores expressed with likelihood ratios. Results demonstrate almost perfect interobserver agreement for 108 glucose dipstick scores (Fleiss’ kappa 0.914) but only moderate interobserver agreement for 96 ketones dipstick scores (Fleiss’ kappa 0.467). Dipstick glucose scores correlated strongly with vitreous humor glucose levels (Spearman’s rank correlation coefficient of 0.841, *n* = 107). Correlation between ketone dipstick scores and serum levels of beta-hydroxybutyrate (BHB) and blood acetone was also positive but much weaker (0.317, *n* = 91; and 0.411, *n* = 92, respectively). The diagnostic value of specific dipstick scores was determined by calculating likelihood ratios for substantial hyperglycemia (vitreous humor glucose > 10 mmol/L), substantial ketoacidosis (serum BHB > 2.50 mmol/L) and elevated blood acetone (> 20 mg/L). Our results suggest substantial screening potential of dipstick urinalysis for glucose, especially when scores are at the lower and higher end of the spectrum. Overall, dipstick analysis results for ketones must be interpreted with great caution. A sub analysis of the data showed that a serum BHB above 2.50 mmol/L was only seen in 1.8% of cases without demonstrable acetone (> 20 mg/L).

## Introduction

The human body requires internal physiological and metabolic stability (or ‘homeostasis’) to survive and function. If homeostasis cannot be maintained, bodily functions can deteriorate rapidly, which may progress into unexpected and/or unexplained death. As such, forensic pathologists are regularly confronted with cases where metabolic and physiological derangements may play an important part in the cause or mechanism of death.

Unfortunately, such derangements are not easy to diagnose in a post-mortem setting [1]. One requires a degree of clinical suspicion to contemplate their presence and their diagnosis relies heavily on ancillary biochemical testing, as they do not necessarily present with abnormalities that are evident on external, internal, histological or radiological examination. The interpretation of post-mortem biochemical analyses furthermore requires knowledge of post-mortem artefacts, whilst considering additional information, such as the deceased’s medical history, the circumstances of death and the decomposition stage.

The usual starting point for post-mortem biochemical analysis is the analysis of vitreous humor (VH) [2], which should be requested in most cases in which no cause of death can be given after autopsy. In selected cases, this analysis may be complemented with testing for ketones (beta-hydroxybutyrate (BHB) and acetone), for instance in vitreous humor, whole blood or serum. These analyses can however be regarded as relatively costly and labor intensive, especially due to the potential need to send samples to specialized biochemical laboratories. Not all mortuaries have access to such facilities, and for those who do, the ancillary testing is usually an additional burden on their budget. The number of cases in which the biochemical analysis proves to be negative for substantial abnormalities is anecdotally high. A screening tool that helps to determine whether biochemical analysis should be performed is therefore desirable.

In a clinical setting, urine dipstick analysis is used as point of care test (POCT) for disorders such as ketosis, diabetes, or urinary tract infections [3]. These dipsticks use absorbent, reactive pads and semi-quantitative color coding to indicate pH, or the concentration of various substances such as glucose, nitrate, bilirubin, ketones, and lymphocytes. They are cheap, easy to use, and provide a result within seconds [4]. The World Health Organization formulated the “ASSURED” criteria for POCTs, indicating that they should be Affordable, Sensitive, Specific, User-friendly, Rapid and Robust, Equipment-free and Deliverable to end users [5]. In theory, urine dipsticks have much potential to also meet these criteria in the post-mortem setting [6]. In a review on the topic of all types of point of care testing (POCT) in the autopsy setting, Ginn et al. noted potential urine dipstick applications, including biochemical and toxicological analyses [7].

Several papers focused on the use of post-mortem dipstick urinalysis for biochemical analysis. One paper was only available in Korean and could not be evaluated in more detail [8]. In a study including 188 forensic autopsies, Mitchell et al. [9] found a sensitivity of 0.83 and specificity of 0.93 for the detection of high glucose vitreous humor levels (≥ 10 mmol/L). For possible ketoacidosis (vitreous BHB ≥ 5 mmol/L) the sensitivity and specificity were 1 and 0.12, respectively. In a similar study including 59 forensic autopsies, Walta et al. found a sensitivity of 0.89 and specificity of 0.90 for elevated glucose levels (≥ 7 mmol/L). The sensitivity and specificity for possible ketoacidosis (vitreous humor total ketones ≥ 3 mmol/L) were 0.84 and 0.68, respectively [10].

Although informative, these studies have shortcomings. For glucose testing, both included many true negative cases (170/188 for Mitchell et al., 50/59 for Walta et al.), which significantly reduced the power of the studies. Furthermore, Walta et al. grouped the dipstick results into positive and negative, diminishing the differentiating power of the dipstick result. For ketones in particular, this considerably increased the number of false negative urine dipstick results. Neither paper tested inter-observer variation. Lastly, neither explored how specific dipstick scores influenced the likelihood of ‘significantly’ elevated glucose or ketones, which would help interpret dipstick scores in individual cases.

Therefore, the aim of this study is to test the validity and diagnostic utility of post-mortem dipstick urinalysis in more detail. More specifically, it determines inter-observer agreement of visual dipstick assessment, as well as the correlation between dipstick scores for glucose and ketones and the results of formal laboratory testing. In addition, the practical value of dipstick urinalysis is determined by calculating likelihood ratios for significant hyperglycemia or ketosis/ketoacidosis for each dipstick score.

Our data furthermore allowed for two sub-analyses. First, whether ancillary BHB testing may still be indicated when the ketone body acetone is not detected on routine toxicology. Second, whether a significant elevation of BHB can be directly inferred from post-mortem blood acetone levels. This may be relevant since acetone can be readily screened for during routine toxicological analysis whilst BHB requires additional specific testing.

## Materials and methods

### Study cohort

This prospective cohort study was performed at the Victorian Institute of Forensic Medicine (VIFM), a statutory body which medically examines the deaths reported to the Victorian Coroner. According to the 2008 Victorian Coroner’s Act [11], this cohort includes all unexplained and unnatural deaths, as well as deaths in certain groups of vulnerable individuals. The VIFM examines approximately 7000 deceased individuals annually, of which approximately one-third are autopsied [12]. This study was approved by the VIFM Research Ethics Committee on 31 August 2023 under application number 1264.

Cases eligible for inclusion were all adult deceased individuals who underwent autopsy at the VIFM, in which the case pathologist requested ancillary investigation for the diagnosis of diabetes mellitus and/or ketosis/ketoacidosis. Cases were excluded where there was visible decomposition, when the autopsy was part of a criminal investigation, or when no post-mortem urine, vitreous humor, or blood samples were available. A total of 108 cases were included.

**Fig. 1:**
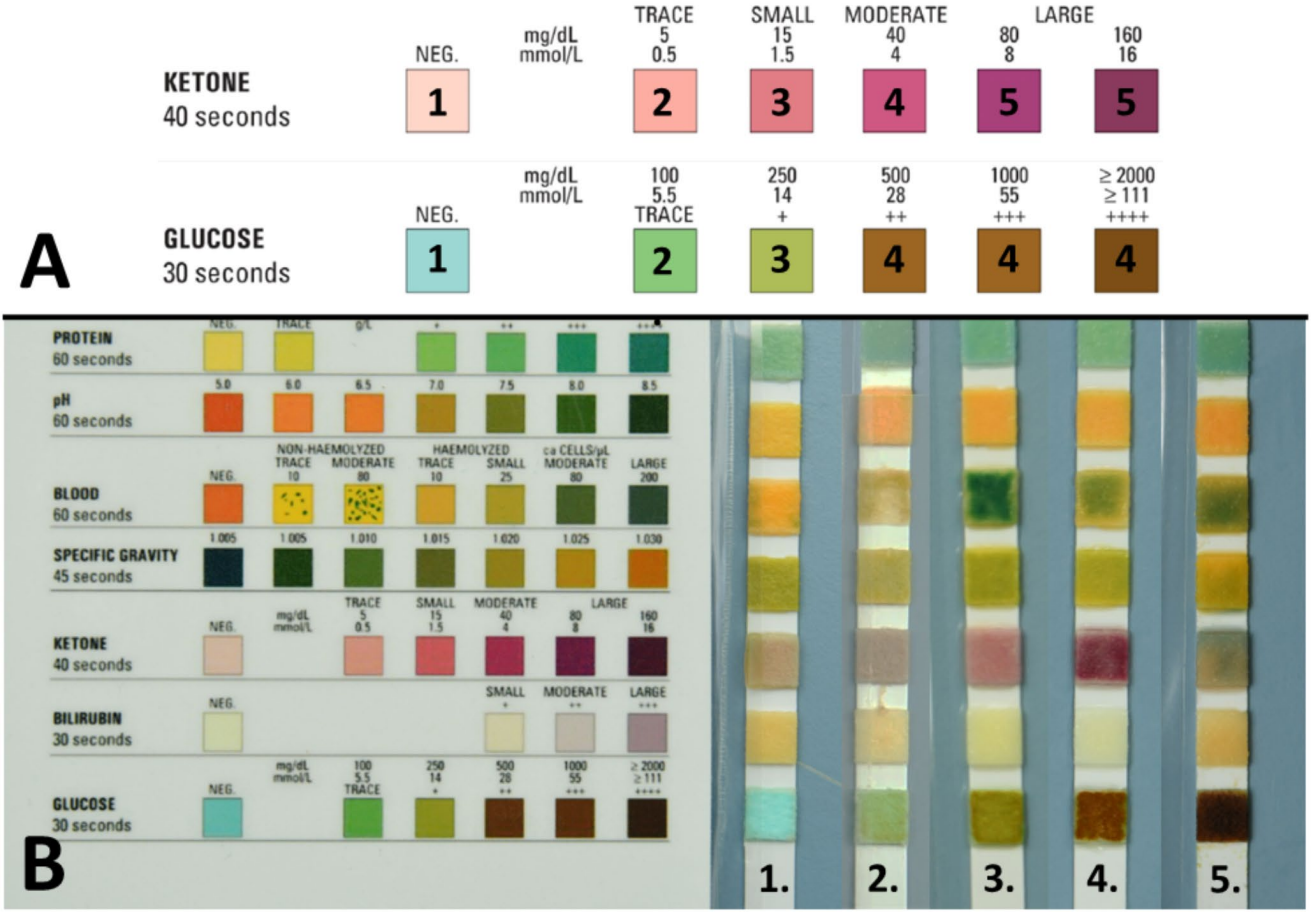
Dipstick scoring sheet with examples. Panel A shows the relevant part of the scoring sheet, demonstrating the numerical categorization of the color scores for this study. Panel B shows various examples of scores. In example 1, glucose and ketones scores are (1) In example 2, glucose and ketone scores are (2) In example 3, glucose and ketone scores are (3) In example 4, glucose score is 4 and ketone score is 5. Example 5 shows a glucose score of 4 and was deemed non-scorable for ketones

For all included cases, urine was sampled from the bladder at autopsy by aspiration with a sterile syringe before bladder opening. The sample was stored in a sterile vial. Dipstick analysis was performed at the time of collection using Livingstone 10SG multiple reagent test strips for urinalysis, in accordance with the manufacturer’s specifications in the package insert. The dipstick was submerged in the urine for 5–10 s, after which it was patted dry. Each dipstick was photographed alongside a laminated version of the visual scoring matrix located on the side of the dipstick container accompanying the dipstick (see Fig. 1 for examples). Two high-quality digital photos were taken to depict the dipstick on a neutral background and adjacent to the score matrix; the first after the 30 s duration required for visual evaluation of glucose scoring and the second 10 s later required for ketone scoring. The photos were stored digitally in the secure proprietary case information management system of the VIFM.

Blood samples from the femoral vein obtained by femoral puncture and aspiration are routinely collected from all deceased admitted to the VIFM. For this research, a blood sample was submitted to the VIFM toxicology laboratory to determine the concentration of the ketone body acetone by gas chromatography with flame ionization detection (in mg/L, with a reporting limit of 20 mg/L). Results below the reporting limit were recorded as negative (0 mg/L) for the purposes of statistical analysis. Another part of the blood sample was centrifuged to obtain a serum sample, of which a portion was transported to the Victorian Clinical Genetics Services, Murdoch Children’s Research Institute to determine BHB serum concentration (in mmol/L).

Vitreous humor samples obtained by needle aspiration are routinely taken during autopsy at the VIFM. For each included case, a sample was submitted to Melbourne Health Pathology (The Royal Melbourne Hospital) for determination of glucose concentration (in mmol/L). Results below the reporting limit of < 0.3 mmol/L were recorded as negative (0.0 mmol/L) for the purposes of statistical analysis.

For each case, the decision to proceed with ancillary biochemical and toxicological analysis was at the discretion of the case pathologist, who had to balance the perceived benefits of additional testing with operational costs and the partial loss of the post-mortem specimen. This resulted in some missing data points that had to be excluded, mostly for serum concentrations of BHB. It however best reflects operational reality.

### Interobserver agreement

Each dipstick photo was presented to five observers who independently scored the dipstick value using the scoring sheet in the photograph (derived from the scoring scale on the side of the dipstick package). Each observer was blinded for the scoring of the other four observers, the autopsy results, and the results of laboratory testing. The possible dipstick scores were categorized numerically, with 1 representing the lowest dipstick score. The two highest scores for ketones (both “large” on the scorecard) and three highest scores for glucose ( + + or higher) were combined into a single score due to their visual similarity and likely equal pathological significance. This resulted in a scoring range of 1–4 for glucose and 1–5 for ketones (See Fig. 1a).

Agreement between the five observers was determined using Fleiss’ kappa [13]. Kappa values were interpreted as suggested by Landis and Koch [14]:

- κ < 0.00: Poor agreement.
- ≤ κ ≤ 0.20: Slight agreement.
- 0.21 ≤ κ ≤ 0.40: Fair agreement.
- 0.41 ≤ κ ≤ 0.60: Moderate agreement.
- 0.61 ≤ κ ≤ 0.80: Substantial agreement.
- 0.81 ≤ κ ≤ 1.00: Almost perfect agreement.

### Correlation analysis between dipstick score and laboratory analysis

Using the average score of the five observers, glucose and ketone dipstick scores were correlated with the results of laboratory testing. For each of the four possible glucose scores, the range, median, mean, and standard error of the mean of vitreous humor glucose concentration were determined. The five possible ketone dipstick scores were correlated in the same way with beta-hydroxybutyrate and acetone testing results. Spearman’s rank correlation coefficient was used to test the strength and direction of the associations, with a p-value < 0.01 indicating statistical significance.

### Diagnostic utility

In ante mortem samples, a fasting blood glucose of 7 mmol/L or greater, or a random blood glucose of 11.1 mmol/L or more in the presence of suggestive symptoms, allows a diagnosis of diabetes mellitus [15]. However, a mere diagnosis of diabetes mellitus is not necessarily relevant for cause and mechanism of death. Also, post-mortem levels are subject to artefact. Cut-off levels used for live patients are therefore not necessarily relevant in a post-mortem setting. There is no one definition of what represents a significantly elevated vitreous glucose level after death, with studies suggesting cut-off values of > 11.1 mmol/L [16], > 7 mmol/L [10], and 13 mmol/L [17]. For our study, we have adopted a cut-off based on Zilg et al. [18], which provides a cogent rationale for using a vitreous glucose cut-off of > 10 mmol/L to indicate that significant hyperglycemia was present prior to death.

In line with many previous publications, a post-mortem BHB serum level of > 2.50 mmol/L was considered ‘forensically significant’ [19]. It has been suggested that acetone concentrations above 90 mg/L can indicate potentially lethal ketoacidosis [20] and other work has indicated that an acetone of approximately 20 mg/L can be used as a cut-off below which ketosis and a significantly elevated BHB can be excluded [21, 22]. As such, we chose 20 mg/L as the level above which the acetone level was considered ‘forensically significant’ in our study cohort of non-decomposed bodies.

The diagnostic utility of the dipstick scores was explored using the above cut-offs and likelihood ratios. The likelihood ratio (LR) framework is the preferred method to evaluate and report evidence in many fields of forensic science [23] and is also advocated in forensic pathology [24]. In a general sense, a LR expresses the extent in which an observation helps to differentiate between two competing and mutually exclusive hypotheses. It is calculated by dividing the probability of the test result under the first hypothesis, divided by the probability of the test result under the competing hypothesis. In the context of this study, LRs were calculated to express the extent in which a glucose dipstick score supports significant hyperglycemia, or a ketone dipstick score supports a potentially relevant ketosis/ketoacidosis. The mutually exclusive hypotheses for these analyses were: VH glucose > 10 mmol/L vs. VH glucose ≤ 10 mmol/L, and serum BHB > 2.50 mmol/L vs. serum BHB ≤ 2.50 mmol/L.

### Association between serum BHB and blood acetone levels

Using the study data, it was furthermore determined whether serum BHB can still be forensically significant (> 2.5 mmol/L) when no acetone was detected on routine toxicology. The potential of bypassing BHB testing was also explored by calculating the likelihood ratio for a BHB serum level of > 2.5 mmol/L vs. BHB serum level ≤ 2.5 mmol/L, for acetone blood levels of 0–20, 20–30, 30–49, and > 100 mg/L.

All statistical analyses were performed with IBM SPSS for Windows (version 29).

## Results

### Inter-observer agreement

For the 108 included glucose dipstick scores, the Fleiss’ Kappa across all scoring categories was 0.914 (95% confidence interval 0.873–0.955) with a significance of < 0.001, meaning that overall, there was almost perfect agreement between the five observers. The agreement was slightly less for a dipstick score of 3. The Fleiss’ kappa values per dipstick score varied slightly and are provided in Table 1.

**Table 1:** Interobserver agreement for post-mortem glucose dipstick scoring

	Obs 1	Obs 2	Obs 3	Obs 4	Obs 5	
Score	*N* (%)	*N* (%)	*N* (%)	*N* (%)	*N* (%)	Kappa (95% CI)
1	39 (36.1)	41 (38)	40 (37)	40 (37)	40 (37)	0.984 (0.924–1.044)
2	10 (9.3)	11 (10.2)	11 (10.2)	12 (11.1)	12 (11.1)	0.811 (0.751–0.871)
3	11 (10.2)	7 (6.5)	8 (7.4)	8 (7.4)	7 (6.5)	0.657 (0.597–0.717)
4	48 (44.4)	49 (45.4)	49 (45.4)	48 (44.4)	49 (45.4)	0.959 (0.899–1.018)
Total	108 (100)	108 (100)	108 (100)	108 (100)	108 (100)	0.914 (0.873–0.955)

Abbreviations Obs = observer; N = number of cases; CI = confidence interval

All Kappa calculations had a p-value of < 0.001

Interobserver agreement in ketone scores was determined for 96 dipsticks. The agreement was moderate with a Fleiss’ kappa of 0.467 (95% confidence interval 0.429–0.504) for the whole cohort. The agreement for separate dipstick scores were moderate for dipstick score 1 to 4 and substantial for score 5. Scores of 4 and 5 were however rarely given (see Table 2) and the results in these categories must therefore be interpreted with caution. One of the 96 dipsticks was especially difficult to score (See example 5 on Fig. 1b), leading to missing data for Observer 1 and Observer 4.

**Table 2:** Interobserver agreement for post-mortem ketone dipstick scoring

	Obs 1	Obs 2	Obs 3	Obs 4	Obs 5	
Score	*N* (%)	*N* (%)	*N* (%)	*N* (%)	*N* (%)	Kappa (95% CI)
1	7 (7.3)	13 (13.4)	10 (10.3)	22 (22.9)	25 (25.8)	0.471 (0.407–0.534)
2	47 (49)	49 (50.5)	42 (43.3)	40 (41.7)	32 (33)	0.475 (0.411–0.538)
3	31 (32.3)	22 (22.7)	18 (18.6)	28 (29.2)	29 (29.9)	0.435 (0.372–0.499)
4	9 (9.4)	11 (11.3)	22 (22.7)	4 (4.2)	8 (8.2)	0.426 (0.363–0.489)
5	2 (2.1)	2 (2.1)	5 (5.2)	2 (2.1)	3 (3.1)	0.742 (0.679–0.806)
Total	96 (100)	97 (100)	97 (100)	96 (100)	97 (100)	0.467 (0.429–0.504)

Abbreviations Obs = observer; N = number of cases; CI = confidence interval

Fleiss’ kappa was calculated for *n* = 96 due to missing data. All Kappa calculations had a p-value of < 0.001

### Correlation with laboratory testing results

Corresponding vitreous humor glucose levels were available for 107 glucose dipstick scores. Results show a clear positive correlation, with vitreous humor biochemistry levels increasing with increasing glucose dipstick scores (See Table 3). This was confirmed with a Spearman’s rank correlation coefficient of 0.841 (p-value < 0.001). There was however considerable overlap in the vitreous humor glucose ranges per dipstick score and significant outliers were noted. In the lowest dipstick score (suggesting no glycosuria), there were two cases with relatively high vitreous humor glucose levels of 8.3 and 6.8 mmol/L. In the highest dipstick score category, there were three cases with a vitreous humor glucose level below 1.0 mmol/L.

**Table 3:** Comparison between post-mortem glucose dipstick result and vitreous humor glucose concentration

		Vitreous humor glucose (mmol/L)			
Dipstick Score	*N*	Range	Median	Mean	SE of mean
1	40	0-8.3	0	0.66	0.26
2	12	0-4.6	0.9	1.37	0.40
3	7	1.1–35.60	10.9	12.13	4.45
4	48	0.3-109.2	35.35	37.26	3.56

Abbreviations N = number of cases; SE = standard error

Corresponding BHB and acetone concentrations were available for 91 and 92 ketone dipstick scores, respectively. Correlation between the ketone dipstick score and BHB concentrations was fair at best, with a Spearman’s rank correlation coefficient of 0.317 (p-value 0.002). Correlation between the ketone dipstick score and acetone concentrations was slightly better (Spearman’s rank correlation coefficient 0.411; p-value < 0.001). Low case numbers for scoring categories 4 (*n* = 9), and 5 (*n* = 2) were again noted.

The overall limited correlation between ketone dipstick scores and laboratory testing was confirmed with the descriptive data presented in Tables 4 and 5. Although BHB and acetone concentrations tend to increase with higher ketone dipstick scores, there is much overlap between the dipstick scores.

**Table 4:** Comparison between post-mortem ketone dipstick results and serum BHB concentration

		Serum beta-hydroxybutyrate (mmol/L)			
Dipstick Score	*N*	Range	Median	Mean	SE of mean
1	13	0.47–22.30	0.36	3.07	1.62
2	43	0.28–19.16	1.43	4.21	0.89
3	24	0.33–16.20	1.48	5.04	1.14
4	9	1.91–38.54	17.29	18.05	3.20
5	2	10.38–11.89	11.14	11.14	0.76

Abbreviations N = number of cases; SE = standard error

**Table 5:** Comparison between post-mortem ketone dipstick results and blood acetone concentration

		Blood acetone (mg/L)			
Dipstick Score	*N*	Range	Median	Mean	SE of mean
1	13	0-410	0	34.50	31.43
2	43	0-530	0	63.42	24.00
3	25	0-800	0	122	42.62
4	9	0-1080	470	500	103.71
5	2	460–470	465	465	5.00

Abbreviations N = number of cases; SE = standard error

### Diagnostic utility expressed with likelihood ratios

The calculated likelihood ratios indicate that glucose dipstick scoring has substantial potential diagnostic utility, especially at the lower and higher end of the spectrum (see Table 6). A low dipstick score (e.g. score 1 or 2) corresponded with LRs of zero, meaning that such a score excluded significant hyperglycemia (> 10 mmol/L) in our cohort. The LR of 12.2 for a dipstick score of 4 indicates that such a result, in our cohort, would increase the pre-test probability of a vitreous humour glucose of > 10 mmol/L approximately twelvefold.

Since ketoacidosis is a known complication of hyperglycemia, it was also calculated whether the glucose dipstick score could be used to directly infer a significantly elevated serum BHB (> 2.5 mmol/L). Glucose dipstick scores of 1–3 had associated likelihood ratios between 0.1 and 0.4, providing weak evidence against a significantly elevated BHB. A glucose dipstick score of 4 doubled the risk of significantly elevated BHB in our cohort (LR of 2.1).

**Table 6:** Likelihood ratios for significant hyperglycemia and ketoacidosis per glucose dipstick result

Dipstick score	Vitreous humor glucose (mmol/L)				Serum beta-hydroxybutyrate (mmol/L)			
	*N*	*N* > 10 (%)	*N* ≤ 10 (%)	LR	*N*	*N* > 2.5 (%)	*N* ≤ 2.5 (%)	LR
1	40	0 (0)	40 (100)	0	34	3 (8.8)	31 (91.2)	0.1
2	12	0 (0)	12 (100)	0	10	2 (20)	8 (80)	0.25
3	7	3 (41.9)	4 (58.1)	0.7	7	2 (28.6)	5 (71.4)	0.4
4	48	41 (85.4)	7 (14.6)	12.2	40	27 (67.5)	13 (32.5)	2.1

Abbreviations N = number of cases; LR = likelihood ratio

Calculated likelihood ratios for the ketone dipstick scores (see Table 7) showed that a dipstick score ≤ 3 argues against significantly elevated BHB serum levels. Based on our data, dipstick scores of 1, 2 and 3 would make such a diagnosis ~ 5 times, ~ 2 times and ~ 1.5 times less likely. A dipstick score of 4 increases the risk of a significant elevation eightfold, whereas a dipstick of 5 excludes a serum BHB **≤** 2.5 mmol/L. However, given the low case numbers in these latter two categories, these likelihood ratios should not be assumed to be representative.

**Table 7:** Likelihood ratios for significant ketoacidosis per ketone dipstick result

Dipstick score		Serum beta-hydroxybutyrate (in mmol/L)		
	*N*	*N* > 2.5 (%)	*N* ≤ 2.5 (%)	LR
1	13	2 (15.3)	11 (84.7)	0.18
2	43	13 (30.2)	30 (69.8)	0.43
3	24	9 (37.5)	15 (62.5)	0.6
4	9	8 (88.9)	1 (11.1)	8
5	2	2 (100)	0 (0)	∞

Abbreviations N = number of cases; LR = likelihood ratio

### Association between serum BHB and blood acetone levels

The cohort had 87 cases in which both BHB and acetone were available. Of those, 55 cases had no measurable acetone blood level, of which only one had a serum BHB level above the threshold of 2.5 mmol/L (2.55 mmol/L). Of the 55 cases with a BHB below 2.5 mmol/L, only one had a measurable blood acetone level (24 mmol/L).

The same data were used to calculate likelihood ratios for significant ketoacidosis, using categories of serum acetone levels (see Table 8). In our cohort, any blood acetone level above 20 mg/L favored a BHB > 2.50 mmol/L. A level between 21 and 50 mg/L increased the probability fourfold, whilst levels above 50 mg/L excluded a BHB level of **≤** 2.50 mmol/L. However, 24 out of the 27 cases in this group had a blood acetone level > 100 mg/L, and only three had a level between 50 and 100 mg/L.

**Table 8:** Likelihood ratios for significant ketoacidosis per blood acetone categories

Blood acetone (mg/L)		Serum beta-hydroxybutyrate (in mmol/L)				
	*N*	Range	Mean	*N* > 2.5 (%)	*N* ≤ 2.5 (%)	LR
0	55	0.28–2.55	1.23	1 (2)	54 (98)	0.02
1–20	0	-	-	-	-	-
21–50	5	1.56–7.49	5.27	4 (80)	1 (20)	4
51–100	3	3.17–4.53	3.96	3 (100)	0 (0)	∞
> 100	24	6.21–38.54	16.26	24 (100)	0 (0)	∞

Abbreviations N = number of cases; LR = likelihood ratio

## Discussion

### Inter-observer agreement

Performing the dipstick test in the mortuary proved to be user-friendly as it was readily available, simple and rapid to perform. In addition, there was almost perfect agreement between observers for glucose scoring, with the highest levels of agreement at the extremes of scoring (scores 1 and 4). This demonstrates that glucose dipstick urinalysis can be readily deployed into practice with confidence that the visual assessment is reliable when applied by different observers.

Ketone scoring proved less reliable, with overall moderate interobserver agreement. There was only substantial agreement for a score of 5, but this was only encountered twice in our cohort. As such, there appear to be substantial issues with the reliability of ketone scoring. The color palette for ketone results has much less variation than the color spectrum for glucose (see Fig. 1b), which makes choosing between the ketone score increments difficult. A scoping internet search suggests this is similar for other brands of urine dipsticks.

Scores were assessed by visualizing a high-quality color image of the dipstick and scoring card on a neutral background, with photographs taken at the 30 and 40-second mark. While this aimed to minimize color variation and provide a consistent and permanent record of the dipstick, artifact or alteration in the appearance of the colors contributing to interobserver variation is not completely excluded.

### Diagnostic utility of post-mortem urine dipstick analysis

The dipstick results were overall positively correlated with the results of formal analyses for vitreous glucose, serum BHB and blood acetone, with all Spearman’s rank correlation coefficient values above zero and statistically significant. For glucose, the correlation was strong, although there was some overlap between VH glucose levels for each dipstick score. Correlation between dipstick ketone score and formal BHB and acetone analysis was only weak and moderate, respectively, with considerable overlap in the blood BHB and acetone levels for each dipstick score.

There were five cases scored for glucose (5/107, 4.7%) that were considered outliers, warranting a closer inspection. Two of these outlier cases had a dipstick score of 1 (suggesting no glycosuria) with relatively high vitreous glucose levels, although these values of 8.6 and 6.8 mmol/L did not meet our threshold of 10 mmol/L for ‘significant hyperglycemia’.

The first case had impaired renal function and died from suicidal sodium nitrite ingestion. Multiple dipsticks showed a consistent glucose score of 1, suggesting the finding was not due to stick failure. Histological analysis showed early decomposition changes, not noted on external examination. Bacterial activity is known to rapidly reduce blood glucose after death [2]. This may have impacted urine levels, whilst the vitreous humor remained relatively shielded from such changes. This possibly reinforces the suggestion that urine dipstick analysis is less accurate when decomposition sets in and suggests that dipstick results may have to re-evaluated when histological evidence of decomposition is noted.

The second case was an elderly individual with longstanding type 2 diabetes mellitus, and hypertensive heart disease, who died from pulmonary infection. These factors, as well as the renal impairment noted in the previous case, may all act to increase the renal tubular threshold for glucose [25], meaning less glucose appears in the urine. This affects the blood glucose at which glycosuria will manifest and therefore may impact the relationship between urine and blood glucose levels. The extent to which this mechanism may have influenced the result in our cohort, however, remains unknown.

Three outliers had a high urine dipstick score of 4 (suggesting a large amount of glycosuria) but vitreous humor glucose levels of less than 1 mmol/L. All three of these individuals were known to have insulin-dependent diabetes. At least two of these individuals were prescribed antidiabetic medication (SGLT2-inhibitors) which may explain the false-positive results. SGLT2 inhibitors are known to produce glycosuria, even in an euglycaemic state. Clinical literature already warned against overinterpretation of urine test results in patients using SGLT2-inhibitors [26]. Our results suggest that SGLT2-inhibitors may produce a positive post-mortem urine glucose dipstick result in euglycaemic individuals. Further research is however needed to corroborate this.

The third individual with a high dipstick score and a low vitreous humour glucose was a type 1 diabetic (not prescribed SGLT2-inhibitors), who died due to a presumed insulin overdose which was unable to be confirmed due to hemolysis in the post-mortem serum samples. The deceased had a considerable amount of urine in their bladder at autopsy. Perhaps in this case, the vitreous humor levels were low due to the effects of excess exogenous insulin, whilst urine acted as a reservoir for more remote glycosuria.

The interpretation of ketone dipstick scoring in relation to the formal BHB and acetone test results requires understanding the mechanics of dipstick testing. A useful exposition on this and the physiology of ketones is provided by Laffel [27]. The major ketone body in ketosis and ketoacidosis is BHB, followed by acetone and acetoacetate. The dipstick, however, only detects acetoacetate and sometimes acetone, not BHB. Therefore, one may miss ketosis or ketoacidosis with low levels of aceto-acetate or acetone, a finding recognized by Gibson et al. [28]. The fact that the acetone and BHB levels are not directly measured by the dipstick may provide an explanation for the only weak to moderate positive correlations between the dipstick score and BHB and acetone levels, and again underscores the limitations of post-mortem dipstick urinalysis for significant ketoacidosis.

Likelihood ratios assess how good a diagnostic test is and help select a proper test or sequence of tests. This is reflected in our data, particularly for glucose. Low dipstick scores (1 or 2) exclude a VH glucose above 10 mmol/L, whilst a dipstick score of 3 is of limited diagnostic value, providing only weak evidence (LR of 0.7) against significant hyperglycemia. A score of 4 indicates that, based on our results, the post-test probability of significant hyperglycemia is approximately twelve times higher than the pre-test probability.

For ketones, the calculated likelihood ratios suggest that dipstick scores of 1, 2 and 3 render a significantly elevated serum BHB approximately 5 times, 2 times and 1.5 times less likely. As such, lower ketone dipstick scores appear to have limited evidential value. Based on the calculated likelihood ratios, high ketone dipstick scores appear to have higher evidential value, with a score of 4 increasing the risk of significantly elevated serum BHB eightfold and all cases with score of 5 having a BHB level of > 2.50 mmol/L. However, given the low case numbers in these categories, these likelihood ratios cannot be assumed to be representative and future research is needed to corroborate our results.

Directly inferring a significant BHB elevation from the glucose dipstick score does little to improve diagnostic certainty. Glucose dipstick scores of 1–3 provide weak evidence (LRs of 0.1–0.4) against a significantly elevated BHB, whilst a score of 4 only doubles its likelihood.

Our data allows a rational basis to interpret most glucose and ketone dipstick scores and for ordering ancillary laboratory testing. Our results are furthermore in line with those of Mitchell et al. and Walta et al., as discussed in the introduction. However, outliers in our cohort, non-conclusive LRs and low case numbers for some categories mean that dipstick results cannot always be considered the final arbiter of whether a metabolic derangement is present. This is particularly relevant when the dipstick findings do not match with the pre-test probabilities assumed by the pathologist. The decision to order ancillary biochemical testing should therefore always take the deceased’s clinical history, clinical presentation, and comorbidities into account.

### The value of serum BHB testing when blood acetone is known

Since Denmark published on the use of BHB in alcoholic ketoacidosis in 1993 [29] there have been conflicting views on whether acetone or BHB are superior biomarkers for ketoacidosis. Since BHB requires additional testing, it would be useful to know whether a negative result for acetone excluded significant BHB levels. The studies of Elliott et al. [30] and Sadones et al. [31] did not reveal any cases with elevated BHB levels without elevated acetone concentrations, and they therefore concluded that BHB testing in absence of measurable acetone is not indicated. Palmiere however, in a comment on the Sadones study, noted that while acetone is a useful marker, BHB is of additional utility [32]. This was borne out in the work of Midtlyng et al. [22] whose 2021 study found 7/376 (1.9%) of their cases had a pathologically significant BHB (> 250 mg/L, equating to approximately 2.4 mmol/L) without detection of acetone in the blood (levels below 23 mg/L).

Our results are in keeping with the results of Midtlyng, since of our 55 cases with no measurable acetone and an available serum BHB level, only one (1.8%) had a significantly elevated BHB level. Death was attributed to diabetic ketoacidosis (glucose was markedly elevated at 34.5 mmol/L) in the setting of concurrent infection. The urine dipstick for ketones detected only a trace, in keeping with the low serum acetone level. Our cohort of known BHB and acetone levels also included one case (0.96%) with an acetone level ≥ 20 mg/L but a BHB < 2.5 mmol/L. This person was not diabetic and glucose was not detected (< 0.3 mmol/L). The autopsy revealed an ischemic cerebral infarct complicated by acute bronchitis and a pulmonary thromboembolism. Cases with a raised acetone and negative BHB (less than 1% of our cases) are difficult to interpret mechanistically but may indicate a degree of ketosis or postmortem formation. Acetone does not contribute to ketoacidosis and its toxicity is limited. Its presence without elevated BHB is therefore unlikely to be of relevance for cause or mechanism of death.

We used likelihood ratios to further explore the relation between serum BHB and blood acetone levels. Serum levels of acetone < 20 mg/L render the probability of a significantly elevated BHB about 50 times less likely. Therefore, acetone testing will be sufficient to identify elevated BHB levels in the vast majority (~ 98%) of cases. In cases with a high clinical suspicion (pre-test probability), BHB testing may still be warranted when acetone levels are negative. Blood acetone levels over 50 mg/L exclude a BHB below 2.5 mmol/L, which appears to make further BHB testing an injudicious endeavor. However, since our cohort only included three cases with a blood acetone level between 50 and 100 mg/L, 100 mg/L may be a safer cut-off value. The relationship between acetone and BHB may differ between diabetic ketoacidosis and other forms of ketoacidosis. In our cohort, a serum BHB of > 2.50 mmol/L was most often diabetic in nature (29 cases) and only 4 times without elevated glucose.

### Limitations and future directions

Some limitations of our study should be noted. First, only test strips of a single brand were used. Most commercially available urinalysis test strips use similar enzymatic reactions for glucose and ketones, and we believe that our results therefore have broader applicability. However, this has not been tested experimentally. Second, as noted before, case numbers for some categories of dipstick scores were low. This was especially the case for glucose scores of 2 and 3, and ketone dipstick scores of 4 and 5. This reflects the relative rarity of these results in daily case work. Our results for these scoring categories should be interpreted with caution and future study is advised to corroborate our results. Availability of data was further impacted by the fact that not all ketone dipstick scores could be correlated with formal BHB testing results. Data was however available in approximately 90% of cases, so the impact on the study findings is expected to be low.

Besides corroboration of our results in a larger cohort, future study may focus on specific post-mortem intervals, to assess whether this affects the correlation between dipstick scoring and laboratory testing is affected before visible decomposition sets in. Validation of urine dipsticks in other matrices than urine (e.g., vitreous humour, cerebrospinal fluid) may also yield interesting results.

## Conclusion

Dipstick urinalysis is overall a rapid and user-friendly screening tool that can support pragmatic and rational decision-making in autopsy cases with (suspected) hyperglycemia and/or ketoacidosis. More specifically, dipstick urinalysis appears to be a reliable and accurate screening tool for significant hyperglycemia, especially when dipstick scores are at the lower or higher end of the spectrum. The relation between dipstick ketone scores and significant ketoacidosis is much less robust, with considerable inter-observer variation and only weak to moderate correlation with formal laboratory test results. This suggests that ketone dipstick results should be interpreted with great caution.

It is important that forensic pathologists do not interpret the results of dipstick urinalysis or ancillary laboratory testing in isolation. Instead, they should evaluate them in the context of the individual case, including case circumstances and patient risk factors. Confirmation of dipstick test results with ancillary biochemical or toxicological analysis is advised.

A sub-analysis showed serum BHB levels above 2.50 mmol/L are rarely seen without detectable blood acetone. Only one such case (1.8%) was present in a cohort of 55 cases with no acetone. Blood acetone levels above 50 mg/L were always associated with a BHB serum level of more than 2.50 mmol/L.

## Data Availability

The data that support the findings of this study are available from the corresponding author upon reasonable request.

## References

[1] Madea B, Musshoff F (2007) Postmortem biochemistry. Forensic Science International, 2007. 165(2–3): 165–171 10.1016/j.forsciint.2006.05.023 16781101

[2] Palmiere C, Mangin P (2012) Postmortem chemistry update part I. Int J Legal Med 126:187–198 21947676 10.1007/s00414-011-0625-y

[3] Simerville JA, Maxted WC, Pahira JJ (2005) Urinalysis: A comprehensive review. Am Family Phys 71(6):1153–1162 15791892

[4] Roberts JR (2007) Urine dipstick testing: everything you need to know. Emerg Med News 29(6):24–27

[5] World Health Organisation (2024) Point-of-care diagnostic tests (POCTS) for sexually transmitted infections (stis). Available from: https://www.who.int/teams/sexual-and-reproductive-health-and-research-(srh)/areas-of-work/sexual-health/sexually-transmitted-infections/point-of-care-tests. Accessed 25 September 2025

[6] Felby S, Nielsen E, Thomsen JL (2008) The postmortem distribution of ketone bodies between blood, vitreous humor, spinal fluid, and urine. Forensic Sci Med Pathol 4:p100–107 10.1007/s12024-007-9018-4 19291479

[7] Ginn C, Ateh D, Martin J (2020) The use of point-of-care testing to Establish cause of death in the autopsy setting. J Forensic Leg Med 71:101933 32342903 10.1016/j.jflm.2020.101933

[8] Lee K, Koo HN, Kim TG et al (2016) Usefulness of dipstick test for vitreous glucose in autopsy practice. Korean J Lab Med 40(4):99–103

[9] Mitchell R, Thomas SD, Langlois NE (2013) How sensitive and specific is Urinalysis ‘dipstick’testing for detection of hyperglycaemia and ketosis? An audit of findings from coronial autopsies. Pathology 45(6):587–590 24018800 10.1097/PAT.0b013e3283650b93

[10] Walta A-M, Keltanen T, Lindroos K et al (2016) The usefulness of point-of-care (poc) tests in screening elevated glucose and ketone body levels postmortem. Forensic Sci Int 266:299–303 27348467 10.1016/j.forsciint.2016.06.003

[11] Coroners Court of Victoria (2008) Coroners Act 2008. Available from: https://www.legislation.vic.gov.au/in-force/acts/coroners-act-2008/042. Accessed on 27 September 2024

[12] Victorian Institute of Forensic Medicine (2023) Annual report 2022–2023. Available from: https://www.parliament.vic.gov.au/491b39/globalassets/tabled-paper-documents/tabled-paper-7957/victorian-institute-of-forensic-medicine-annual-report-2022-23.pdf. Accessed on 27 September 2024

[13] Fleiss JL, Levin B, Paik MC (2013) Statistical methods for rates and proportions. John Wiley & Sons Inc., Hoboken, New Jersey

[14] Landis JR, Koch GG (1977) The measurement of observer agreement for categorical data. Biometrics 33(1):159–174 843571

[15] World Health Organization (2019) Classification of diabetes mellitus. World Health Organization: Geneva. ISBN 978-92-4-151570-2

[16] Coe JI (1993) Postmortem chemistry update emphasis on forensic application. Am J Forensic Med Pathol 14(2):91–117 8328447 10.1097/00000433-199306000-00001

[17] Karlovsek M (2004) Diagnostic values of combined glucose and lactate values in cerebrospinal fluid and vitreous humour—our experiences. Forensic Sci Int 146:S19–S23 15639573 10.1016/j.forsciint.2004.09.006

[18] Zilg B, Alkass K, Berg S et al (2009) Postmortem identification of hyperglycemia. Forensic Sci Int 185(1–3):89–95 19167848 10.1016/j.forsciint.2008.12.017

[19] Palmiere C, Mangin P (2012) Postmortem chemistry update part II. Int J Legal Med 126:199–215 21984165 10.1007/s00414-011-0614-1

[20] Brinkmann B, Fechner G, Karger B (1998) Ketoacidosis and lactic acidosis–frequent causes of death in chronic alcoholics? Int J Legal Med 111:115–119 9587792 10.1007/s004140050130

[21] Hydara YE, Zilg B (2020) Postmortem diagnosis of ketoacidosis: levels of beta-hydroxybutyrate, acetone and isopropanol in different causes of death. Forensic Sci Int 314:110418 32711386 10.1016/j.forsciint.2020.110418

[22] Midtlyng L, Høiseth G, Luytkis H et al (2021) Relationship between betahydroxybutyrate (BHB) and acetone concentrations in postmortem blood and cause of death. Forensic Sci Int 321:110726 33631622 10.1016/j.forsciint.2021.110726

[23] Nordgaard A, Rasmusson B (2012) The likelihood ratio as value of evidence—more than a question of numbers. Law Probab Risk 11(4):303–315

[24] De Boer HH, Fronczek J, Berger CE et al (2022) The logic of forensic pathology opinion. Int J Legal Med 136(4):1027–1036 34988615 10.1007/s00414-021-02754-1

[25] Hieshima K, Sugiyama S, Yoshida A et al (2020) Elevation of the renal threshold for glucose is associated with insulin resistance and higher glycated hemoglobin levels. J Diabetes Invest 11(3):617–625 10.1111/jdi.13191 PMC7232275 31770476

[26] Schwartz AL (2024) SGLT2 inhibitors and false positive toxicology tests. N Engl J Med 390(6):573–574 38324493 10.1056/NEJMc2313463 PMC11665753

[27] Laffel L (1999) Ketone bodies: A review of physiology, pathophysiology and application of monitoring to diabetes. Diab/Metab Res Rev 15(6):412–426 10.1002/(sici)1520-7560(199911/12)15:6<412::aid-dmrr72>3.0.co;2-8 10634967

[28] Gibson AA, Eroglu EI, Rooney K et al (2020) Urine dipsticks are not accurate for detecting mild ketosis during a severely energy restricted diet. Obes Sci Pract 6(5):544–551 33082996 10.1002/osp4.432 PMC7556427

[29] Denmark LN (1993) The investigation of beta-hydroxybutyrate as a marker for sudden death due to hypoglycemia in alcoholics. Forensic Sci Int 62(3):225–232 8307532 10.1016/0379-0738(93)90211-r

[30] Elliott S, Smith C, Cassidy D (2010) The post-mortem relationship between beta-hydroxybutyrate (BHB), acetone and ethanol in ketoacidosis. Forensic Sci Int 198(1–3):53–57 19954904 10.1016/j.forsciint.2009.10.019

[31] Sadones N, Lambert WE, Stove CP (2017) The (non) sense of routinely analysing beta-hydroxybutyric acid in forensic toxicology casework. Forensic Sci Int 274:38–43 28089299 10.1016/j.forsciint.2017.01.002

[32] Palmiere C (2017) The (non) sense of routinely analyzing beta-hydroxybutyrate in forensic toxicology casework. Forensic Sci Int 279:e18–e19 28318652 10.1016/j.forsciint.2017.02.021

